# Integrated Bioinformatics Analysis of Hub Genes and Pathways in Anaplastic Thyroid Carcinomas

**DOI:** 10.1155/2019/9651380

**Published:** 2019-01-13

**Authors:** Xueren Gao, Jianguo Wang, Shulong Zhang

**Affiliations:** ^1^Department of Pediatric Endocrinology/Genetics, Xinhua Hospital, School of Medicine, Shanghai Jiao Tong University, Shanghai 200092, China; ^2^Department of General Surgery, Xuhui District Central Hospital of Shanghai, Shanghai 200031, China

## Abstract

Anaplastic thyroid carcinoma (ATC) is a very rare malignancy; the pathogenesis of which is still not fully understood. The aim of the present study was to identify hub genes and pathways in ATC by microarray expression profiling. Two independent datasets (GSE27155 and GSE53072) were downloaded from GEO database. The differentially expressed genes (DEGs) between ATC tissues and normal thyroid tissues were screened out by the limma package and then enriched by gene ontology (GO) and KEGG pathway analysis. The hub genes were selected by protein-protein interaction (PPI) analysis. A total of 141 common upregulated and 87 common downregulated genes were screened out. These DEGs were significantly enriched in the phagosome and NF-kappa B signaling pathway. Through PPI analysis, *TOP2A*, *TYMS*, *CCNB1*, *RACGAP1*, *FEN1*, *PRC1*, and *UBE2C* were selected as hub genes, which were highly expressed in ATC tissues. TCGA data suggested that the expression levels of *TOP2A*, *TYMS*, *FEN1*, and *PRC1* genes were also upregulated in other histological subtypes of thyroid carcinoma. High expression of *TOP2A*, *TYMS*, *FEN1*, *PRC1*, or *UBE2C* gene significantly decreased disease-free survival of patients with other thyroid carcinomas. In conclusion, the present study identified several hub genes and pathways, which will contribute to elucidating the pathogenesis of ATC and providing therapeutic targets for ATC.

## 1. Introduction

Thyroid carcinoma is a common endocrine cancer accounting for approximately 1.7% of total cancer diagnoses [1]. The main histologic types of thyroid carcinoma include papillary thyroid carcinoma (PTC), follicular thyroid carcinoma, medullary thyroid carcinoma, and anaplastic thyroid carcinoma (ATC). Approximately 80% of thyroid cancers are PTCs, which are usually curable with a 5-year survival of over 95% [2]. In contrast, ATC constitutes only a small part (1–2%) of all thyroid carcinomas, but it is the most malignant with a median survival of 3–5 months [3, 4]. To date, there is no standard or effective therapy for ATC. Thus, it is very urgent to understand the pathogenesis of ATC, which will contribute to the discovery of the attractive therapeutic targets.

In the past decades, traditional and molecular biological techniques have been used to reveal ATC-related genes and pathways [5–7]. For instances, Yin et al. found that the downregulated expression of the forkhead box D3 (FOXD3) transcription factor in ATC cells promoted invasiveness and epithelial-to-mesenchymal transition (EMT) and decreased cellular apoptosis. FOXD3 silencing also enhanced p-ERK levels in the ATC cells, suggesting it negatively regulated MAPK/ERK signaling [5]. Zhang et al. observed that S100A4 was highly expressed in ATC tissues. Knockdown of S100A4 significantly decreased proliferation, increased apoptosis, and inhibited the invasive potential of ATC cells [6]. Salerno et al. found that TWIST1 played a pleiotropic role in determining the ATC phenotype. The ectopic expression of TWIST1 induced resistance to apoptosis and increased cellular migration and invasion [7]. Although major efforts to clarify the pathogenesis of ATC are ongoing, the relevant progress is still not obvious. Considering the complexity and heterogeneity of ATC, we adopted microarray technology and bioinformatics methods to systematically explore large-cohort gene expression in ATC tissues, which has been demonstrated to be valuable for molecular mechanism investigation [8].

## 2. Materials and Methods

### 2.1. Microarray Data

The raw data of gene expression profiles of 9 ATC and 7 normal thyroid tissues were downloaded from Gene Expression Omnibus database (GEO accession numbers: GSE27155 and GSE53072). GSE27155 and GSE53072 datasets were submitted by Giordano et al. and Pita et al., respectively [9–11]. Among these analyzed tissues, 4 ATC and 4 normal thyroid tissues from GSE27155 were detected by Affymetrix Human Genome U133A Array (GPL96 platform). Other tissues from GSE53072 were detected by Affymetrix Human Gene 1.0 ST Array (GPL6244 platform).

The raw data of GSE27155 and GSE53072 were preprocessed using the affy and oligo packages with the robust multichip averaging (RMA) algorithm, respectively [12–16]. The probeset IDs were converted into the corresponding gene symbols using the annotation information derived from platforms. If multiple probesets correspond to the same gene, the mean expression values of those probesets were obtained.

### 2.2. Identification of Differentially Expressed Genes (DEGs)

Limma R package was applied to identify the DEGs between ATC and normal thyroid tissues [17]. The Benjamini-Hochberg (BH) method was introduced to adjust the raw *p* values. The adjusted *p* value < 0.05 and |log2 fold change (FC)| ≥ 1 were set as the thresholds for identifying DEGs.

### 2.3. Functional and Pathway Enrichment Analysis of DEGs

In order to systematically explore genes involved in ATC, gene ontology (GO) and pathway enrichment analyses for the common DEGs were performed using the clusterProfiler package in R, which was based on the GO and Kyoto Encyclopedia of Genes and Genomes (KEGG) databases [18–20]. The criterion for the significant enrichments was set as *p* value < 0.05.

### 2.4. Construction of the Protein-Protein Interaction (PPI) Network

The PPI network provides a valuable framework for better understanding of the functional organization of the proteome. In this network, nodes and edges represent proteins and interactions between proteins, respectively. The proteins with high degrees, namely, those highly connected with other proteins, are defined to be located at the network center, which may be regulatory “hubs.” We constructed the PPI network by using STRING database and Cytoscape 3.3 software [21, 22]. The nodes with degree > 1 were reserved in the PPI network. Genes with degree > 25 were considered as hub genes (proteins).

### 2.5. The Expression Levels of Hub Genes in Other Thyroid Carcinomas

UALCAN (http://ualcan.path.uab.edu) is a user-friendly, interactive web resource for analyzing cancer transcriptome data from The Cancer Genome Atlas (TCGA) [23]. In the current study, UALCAN was used to explore the expression levels of hub genes in other thyroid carcinomas. *p* value < 0.05 was considered statistically significant.

### 2.6. The Association of Hub Gene Expression with Disease-Free Survival of Patients with Other Thyroid Carcinomas

Gene expression profiling interactive analysis (GEPIA, http://gepia.cancer-pku.cn/) is a web-based tool to deliver fast and customizable functionalities based on TCGA and GTEx data [24]. These functionalities included differential expression analysis, profiling plotting, correlation analysis, patient survival analysis, similar gene detection, and dimensionality reduction analysis. In the current study, GEPIA was used to explore the association of hub gene expression with disease-free survival of patients with other thyroid carcinomas. Patients were grouped into high expression group and low expression group according to the median value of hub gene expression. *p* value < 0.05 was considered statistically significant.

## 3. Results

### 3.1. Identification of DEGs

**Figure 1:**
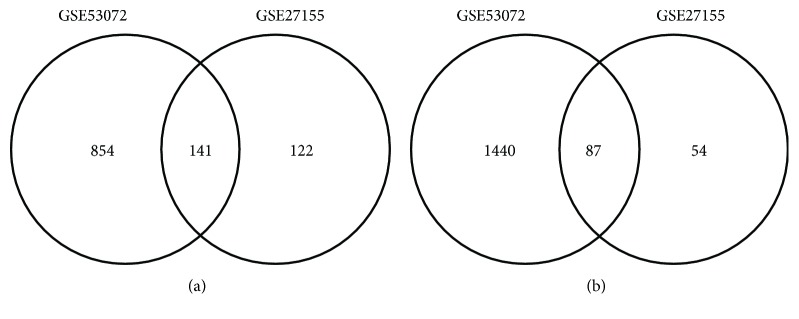
Venn diagrams showing the number of differentially expressed genes (DEGs) in ATC tissues compared with normal thyroid tissues ((a) the number of upregulated genes; (b) the number of downregulated genes).

As shown in Figure 1 and Table 1, for GSE27155, a total of 404 DEGs, including 263 upregulated and 141 downregulated genes in ATC, were identified. For GSE53072, a total of 2522 DEGs, including 995 upregulated and 1527 downregulated genes in ATC, were screened out. Further analysis showed that the two independent datasets contained 228 common DEGs, including 141 common upregulated and 87 common downregulated genes in ATC.

### 3.2. Integrated Analysis of the Common DEGs

**Figure 2:**
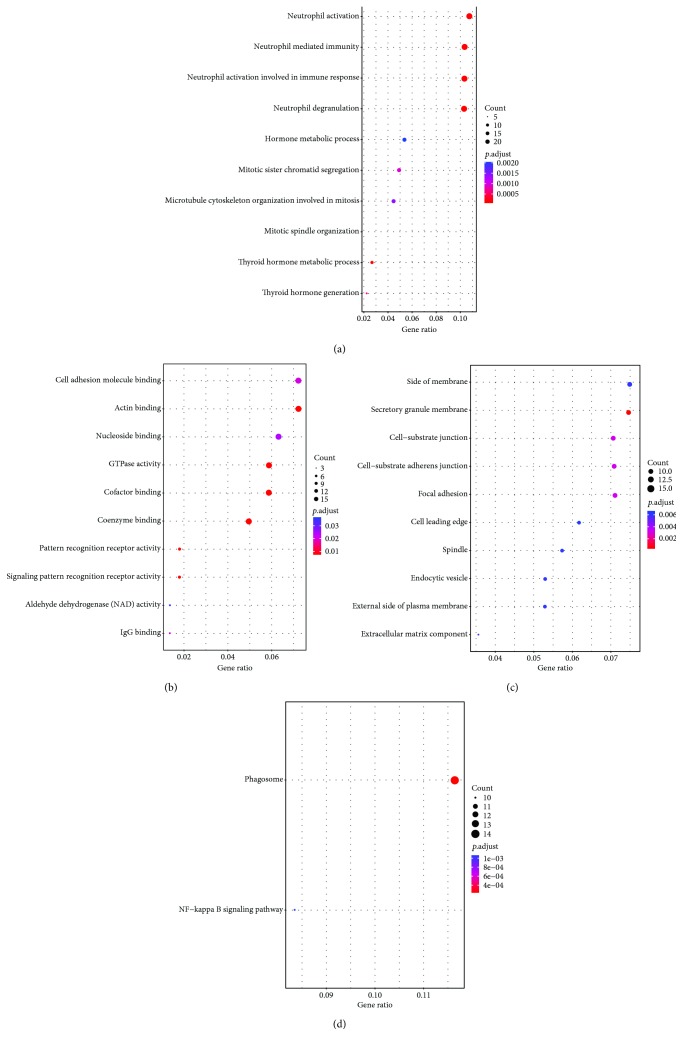
The top 10 gene ontology (GO) terms and significantly enriched KEGG pathways ((a) biological processes; (b) molecular functions; (c) cellular components; (d) KEGG pathways).

By GO analysis, we identified 146 significant enrichments terms, which were classified in 3 GO categories, including biological processes (BP, 92), molecular functions (MF, 20), and cellular components (CC, 34). The top 10 GO terms of BP, MF, and CC were shown in Figures 2(a)–2(c). For BP, those common DEGs are significantly enriched in neutrophil activation, neutrophil-mediated immunity, neutrophil activation involved in immune response, neutrophil degranulation, hormone metabolic process, mitotic sister chromatid segregation, microtubule cytoskeleton organization involved in mitosis, mitotic spindle organization, thyroid hormone metabolic process, thyroid hormone generation, and so on. For MF, those common DEGs are mainly enriched in cell adhesion molecule binding, actin binding, nucleoside binding, GTPase activity, cofactor binding, coenzyme binding, pattern recognition receptor activity, signaling pattern recognition receptor activity, aldehyde dehydrogenase (NAD) activity, and IgG binding. For CC, those common DEGs are significantly enriched in the side of the membrane, secretory granule membrane, cell-substrate junction, cell-substrate adherens junction, focal adhesion, cell leading edge, spindle, endocytic vesicle, external side of plasma membrane, extracellular matrix component, and so on. Furthermore, KEGG pathway enrichment analysis showed that these common DEGs were significantly enriched in the phagosome and NF-kappa B signaling pathway (Figure 2(d)).

**Figure 3:**
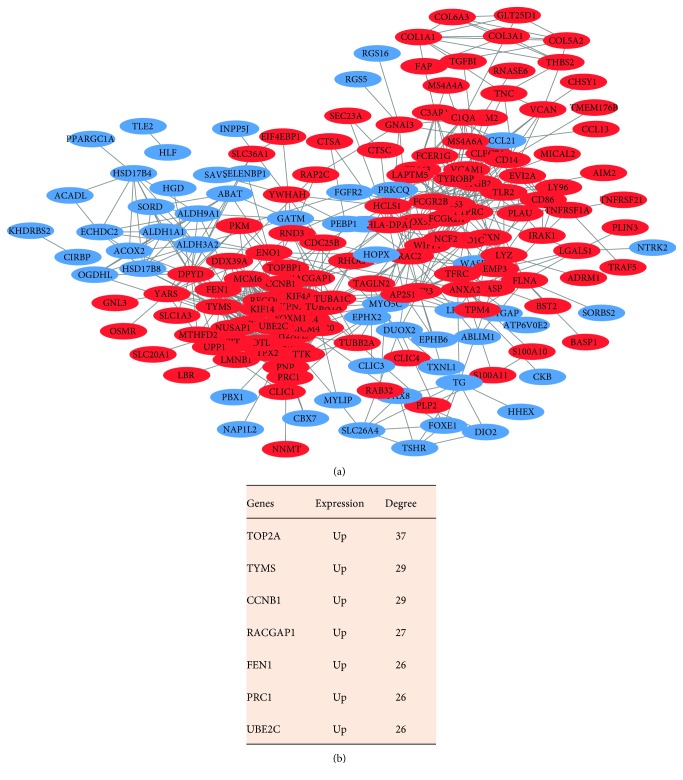
Protein-protein interaction (PPI) network of differentially expressed genes (DEGs) ((a) PPI network; (b) hub genes).

There were 180 nodes and 737 edges in the PPI network (Figure 3(a)). Thereinto, 7 upregulated genes in ATC, including *TOP2A*, *TYMS*, *CCNB1*, *RACGAP1*, *FEN1*, PRC1, and UBE2C, were selected as the hub genes (Figure 3(b)). *TOP2A* gene had the highest degree (degree = 37) in the network.

**Figure 4:**
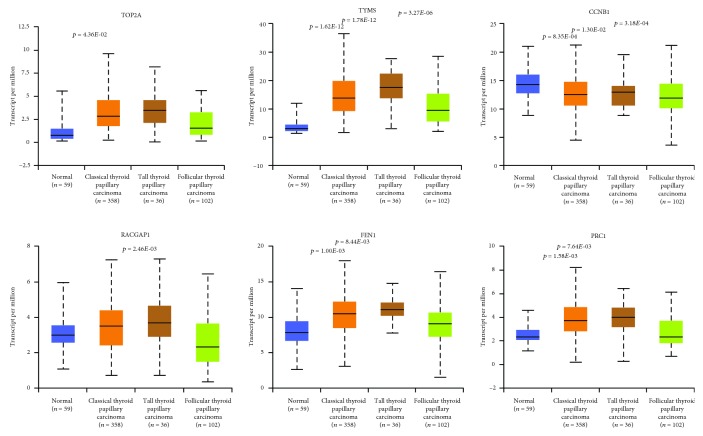
The expression levels of hub genes in other thyroid carcinomas.

**Figure 5:**
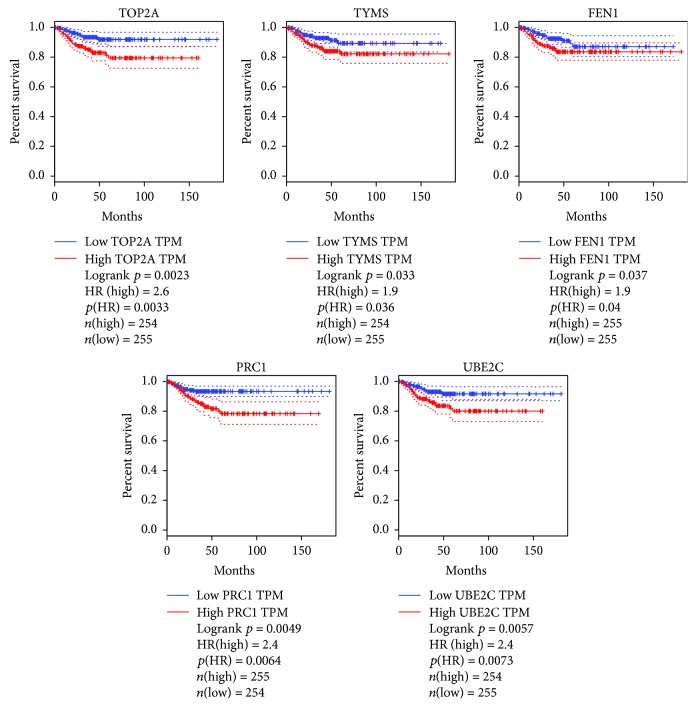
The association of hub gene expression with disease-free survival of patients with other thyroid carcinomas.

UALCAN and GEPIA online tools were used to explore TCGA data. Results suggested that the expression levels of hub genes, *TOP2A*, *TYMS*, *FEN1*, and *PRC1*, were also upregulated in at least one histological subtype of thyroid carcinoma (Figure 4). However, the expression levels of *CCNB1* and *RACGAP1* genes were significantly downregulated in at least one histological subtype of thyroid carcinoma. *UBE2C* gene expression did not significantly change in other thyroid carcinomas (figure not shown). Survival analysis indicated that high expression of *TOP2A*, *TYMS*, *FEN1*, *PRC1*, or *UBE2C* gene significantly decreased disease-free survival of patients with other thyroid carcinomas (Figure 5).

## 4. Discussion

In the present study, we identified two significant pathways (phagosome and NF-kappa B signaling pathway) and seven hub genes (*TOP2A*, *TYMS*, *CCNB1*, *RACGAP1*, *FEN1*, *PRC1*, and *UBE2C*) related with ATC. In 2017, Huang et al. used GSE33630 data to conduct a similar analysis and considered the Toll-like receptor signaling pathway, extracellular matrix-receptor interaction, and cytokine-cytokine receptor interaction pathway as important pathways implicating ATC. *FOS*, *CXCL10*, *COL5A1*, *COL11A1*, and *CCL28* genes might be used as therapeutic targets for ATC [25]. These findings were different from our findings, which might be attributed to tumor heterogeneity or differences in analytical methods between two studies. Besides the identification of potential hub genes and pathways associated with ACT, we still analyzed the expression levels of these hub genes in other histological subtypes of thyroid carcinoma and found that UBE2C gene expression did not significantly change in other thyroid carcinomas, suggesting that *UBE2C* might act as a specific diagnostic biomarker for ACT. Further survival analysis showed that high expression of *TOP2A*, *TYMS*, *FEN1*, *PRC1*, or *UBE2C* gene significantly decreased disease-free survival of patients with other thyroid carcinomas.

Although there was no study directly linking phagosome to ATC, the phagosome participated in the innate and adaptive immune responses [26, 27]. The present study found ten common DEGs involving in the phagosome. Therefore, the role of the phagosome and phagosome-related genes in ATC was worthy of being further explored. NF-kappa B signaling pathway played an important role in cancer initiation and progression [28, 29]. The present analysis of DEGs showed that most genes of the NF-kappa B signaling pathway were upregulated, suggesting that the pathway was also activated in ATC. Furthermore, the NF-kappa B signaling pathway also participated in an anticancer agent (R-roscovitine) activity, inducing apoptosis of ATC cells [30]. These results indicated that novel agents involving the NF-kappa B signaling pathway could be developed to improve ATC treatment.

Previous studies have indicated that seven hub genes identified by the present study play an important role in the occurrence and development of tumors [31–46]. *TOP2A* gene encodes a DNA topoisomerase that controls and alters the topologic states of DNA during transcription. Immunohistochemical analysis showed that TOP2A correlated with thyroid tumor histology and it was more frequently expressed in tumors with aggressive clinical behavior [31]. *TYMS* gene encodes thymidylate synthase, which catalyzes the methylation of deoxyuridylate to deoxythymidylate. Although the role of TYMS in ATC was not reported previously, the enzyme has been of interest as a target for cancer chemotherapeutic agents [32–35]. The protein encoded by *CCNB1* gene is involved in mitosis and necessary for proper control of the G2/M transition phase of the cell cycle. Lin et al. found that dinaciclib, a cyclin-dependent kinase (CDK) inhibitor, could inhibit ATC cell proliferation by decreasing CDK1, CCNB1, and Aurora A expression, inducing cell cycle arrest in the G2/M phase and inducing the accumulation of prophase mitotic cells [36]. *RACGAP1* gene encodes a GTPase-activating protein (GAP) that is a component of the centralspindlin complex. This protein played a regulatory role in cytokinesis, cell growth, and differentiation. To date, there is no report linking RACGAP1 to ATC, but its importance in the development of other cancers has been revealed [37, 38]. The protein encoded by *FEN1* gene removes 5′ overhanging flaps in DNA repair and processes the 5′ ends of Okazaki fragments in lagging strand DNA synthesis. Previous studies confirmed that FEN1 not only promoted cancer cell proliferation and progression but also conferred cancer drug resistance [39, 40]. According to these results, we inferred that FEN1 might also play important roles in development or drug resistance of ATC. *PRC1* gene encodes a protein involved in cytokinesis [41, 42]. The protein is present at high levels during the S and G2/M phases of mitosis, but its levels drop dramatically when the cell exits mitosis and enters the G1 phase. Recent studies showed that PRC1 was upregulated in many types of cancer and might serve as a prognostic biomarker of cancer [43–45]. *UBE2C* gene encodes a member of the E2 ubiquitin-conjugating enzyme family. The encoded protein is required for the destruction of mitotic cyclins and for cell cycle progression. Pallante et al. found that UBE2C overexpression was involved in thyroid cell proliferation and might act as a diagnostic biomarker for ATC [46].

Taken together, the integrated bioinformatics study presented several hub genes and pathways related to ATC, which would provide new insights into the exploration of pathogenesis and therapeutic targets for ATC.

## Figures and Tables

**Table 1:** The common differentially expressed genes in GSE27155 and GSE53072 datasets. Note: bold and italic data indicate genes in the phagosome and NF-kappa B signaling pathway, respectively. Bold-italic data indicates the common gene in the phagosome and NF-kappa B signaling pathway.

Common differentially expressed genes										
Downregulated genes				Upregulated genes						
MAN1C1	CA4	WASF3	HSD17B4	C1QA	**NCF2**	**CLEC7A**	RAB31	COL5A2	CCNB1	RAP2C
PBX1	KCNJ16	CLN5	MYLIP	CLIC4	ASPM	RECQL	PMAIP1	COL6A3	VCAN	*IRAKI*
Clorfll5	PTPRM	RCBTB1	HSD17B8	CDC20	KIF14	**TUBA1A**	ADGRE5	CDC25B	LMNB1	FLNA
MARC2	TXNL1	TSHR	MTCH1	*VCAM1*	LBR	RACGAP1	TPM4	TPX2	TGFBI	TAGLN2
MARC1	CIRBP	SAV1	KHDRBS2	GPSM2	ARID5B	CORO1C	COLGALT1	UBE2C	SLC36A1	C3AR1
RAP1GAP	BCAM	DI02	LMBRD1	GNAI3	*PLAU*	CIT	VASP	CTSA	LHFPL2	TYMS
ECHDC2	TLE2	CKB	SLC26A4	CD53	ZWINT	ALOX5AP	EMP3	ADRM1	***CD14***	WIPF1
PLPP3	PAX8	PPP1R13B	EPHB6	IFI16	MICAL2	PNP	PLIN3	B4GALT5	TTK	OSMR
SELENBP1	LRP2	SORD	**ATP6V0E2**	FCER1G	MS4A4A	RNASE6	DDX39A	**ITGB2**	RAB32	KIF4A
RGS5	ACADL	DUOX1	TMEM243	**FCGR2A**	FEN1	BAZ1A	BST2	YWHAH	**TUBB2A**	
ALDH9A1	RAB17	CRABP1	EPHX2	**FCGR2B**	NNMT	SEC23A	TYROBP	LGALS1	CLIC1	
RGS16	SNTA1	DUOX2	TG	PTPRC	RHOG	NUSAP1	AP2S1	RAC2	**HLA-DPA1**	
HHEX	INPP5J	GATM	TJP2	*TRAF5*	LPXN	ANXA2	RRM2	GNL3	TNFRSF21	
*PRKCQ*	CBX7	MY05C	NTRK2	DTL	MS4A6A	PKM	MTHFD2	CD86	**THBS2**	
NEBL	ACOX2	DAPK2	FOXE1	ENOl	PPP1R14B	PRC1	TMSB10	SMC4	UPP1	
OGDHL	ZBED2	ABAT	BSPRY	LAPTM5	Cl lorf24	CHSY1	SLC20A1	HCLS1	TMEM176B	
ABLIM1	HGD	SALL1	*CCL21*	YARS	CTSC	*CCL13*	ACTR3	TOPBP1	EIF4EBP1	
FGFR2	LIMCH1	CDH16	ALDH1A1	DPYD	**TUBA1C**	KPNA2	COL3A1	**TFRC**	MCM4	
GLB1L2	PPARGC1A	ALDH3A2	CLIC3	MTMR11	LYZ	SLC16A3	PXDN	**TLR2**	*LY96*	
METTL7A	HOPX	DUSP14	RGN	S100A10	FOXM1	EVI2A	MCM6	H2AFZ	ANXA2P2	
PEBP1	SORBS2	HLF	GPRASP1	S100A11	*TNFRSF1A*	TOP2A	RND3	BASP1	TNC	
IFT88	PDE8B	NAP1L2		AIM2	CD163	COL1A1	FAP	SLC1A3	PLP2	

## Data Availability

The microarray data used to support the findings of this study have been deposited in the Gene Expression Omnibus (GEO) repository (accession numbers: GSE27155 and GSE53072).
